# Proliferative diabetic retinopathy without preoperative pan-retinal photocoagulation is associated with higher levels of intravitreal IL-6 and postoperative inflammation

**DOI:** 10.1186/s40942-020-00222-3

**Published:** 2020-06-08

**Authors:** Yukihiko Suzuki, Kobu Adachi, Natsuki Maeda, Reiko Tanabu, Takashi Kudo, Mitsuru Nakazawa

**Affiliations:** Department of Ophthalmology, Hirosaki University Graduate School of Medicine, 5 Zaifu, Hirosaki, Aomori 036-8562 Japan

**Keywords:** Diabetic retinopathy, Vitrectomy, Interleukin-6, Vascular endothelial growth factor

## Abstract

**Purpose:**

Intravitreal cytokine levels and differences in the severity of postoperative inflammation in patients with proliferative diabetic retinopathy (PDR) were compared between subjects treated with pan-retinal photocoagulation (PRP) prior to undergoing vitreous surgery and those treated similarly during the surgery.

**Subjects and methods:**

We examined 130 eyes of PDR patients who underwent an initial surgical procedure at Hirosaki University Hospital. A total of 67 out of the 130 eyes were treated with PRP prior to surgery (preoperative group), while 63 underwent similar treatment during surgery (intraoperative group). Vitreous fluid was collected at the start of the vitreous surgical procedure. Following vitrectomy, intraoperative PRP was completed during the surgery in the intraoperative group. This procedure was added to the peripheral part as needed in the preoperative group. The results for the levels of 27 cytokines, including vascular endothelial growth factor (VEGF), were measured using multiplex assays and compared between the groups. For determining postoperative inflammation, eyes in which fibrin was observed for 3 days or longer in the anterior chamber or vitreous cavity were considered to have fibrin deposition. The rate of fibrin deposition was compared between these groups.

**Results:**

Among the vitreous cytokines examined, there was no significant difference in the VEGF levels between the groups (p = 0.70). In contrast, the IL-6 level was significantly higher in the intraoperative group (2813.2 ± 6022.2 vs. 1248.5 ± 1442.2 pg/ml, p = 0.048). Furthermore, the fibrin deposition rate was significantly higher in the intraoperative (44.4%) versus the preoperative group (14.9%) (p = 0.0002).

**Conclusion:**

Severe postoperative inflammation was frequently seen in patients who did not undergo preoperative PRP, which may have been due to the performance of the procedure during the surgery. Moreover, these results may also be associated with a higher level of intravitreal IL-6.

## Introduction

In conjunction with an increasing number of patients with type 2 diabetes, diabetic retinopathy (DR) has now become a major health issue worldwide. For treatment of type 2 diabetes, although intensive glycemic control has been reported to be effective in both reducing the risk of microvascular complications and the need for retinal photocoagulation [1], strict blood pressure regulation is also known to prevent microvascular complications [2].

Retinal photocoagulation, especially pan-retinal photocoagulation (PRP), has been extensively used to treat patients with diabetic retinopathy in order to prevent progression or avoid resultant loss of vision [3, 4]. Recently, some studies have found that PRP combined with a retinal injection of an anti-vascular endothelial growth factor (VEGF) agent was more effective than a single application of PRP [5] and that patients treated with photocoagulation combined with triamcinolone acetonide [6] had fewer macular edema postoperative complications. As a result, studies have been investigating the importance of photocoagulation, as well as improvements in therapy related to treatments associated with intraocular VEGF and inflammatory cytokines.

It has been suggested that levels of intravitreal interleukin (IL)-6 [7–10], IL-8 [8, 10, 11], TNF-α [7, 9, 12], IL-1β [10], VEGF [8, 13–15], intercellular adhesion molecule (ICAM)-1 [15], and monocyte chemotactic protein (MCP)-1 [8, 16] are higher in patients with proliferative DR (PDR) and/or diabetic macular edema. It is known that VEGF, which is associated with retinal ischemia, can induce retinal angiogenesis and increase vascular permeability. Furthermore, it has also been reported that the severity of DR is related to the level of intravitreal VEGF. Thus, inflammatory cytokines such as IL-6 in addition to VEGF, which increase due to retinal ischemia, may be associated with DR progression. Moreover, it has also been suggested that PRP might prevent this advancement of DR [3, 17, 18].

In addition, it has yet to be determined whether differences in levels of intravitreal cytokines related to the severity of postoperative inflammation exist between patients who have undergone a vitrectomy after PRP and those who received surgery without prior PRP.

### Subjects and methods

The present study was performed in accordance with the tenets of the Declaration of Helsinki and approved by the Institutional Review Board of Hirosaki University Graduate School of Medicine (approval number: 2014-353, UMIN: 000022734). Written informed consent was obtained from each patient after receiving an explanation of the nature and possible consequences of taking part in the study.

### Patients

We examined 130 eyes in 106 PDR patients (type 2 diabetes) who underwent an initial vitrectomy performed by one of the authors (Y.S.) in the Department of Ophthalmology at the Hirosaki University School of Medicine Hospital between September 2010 to August 2012.

Retinal conditions were classified into four grades: neovascularization (NV), vitreous hemorrhage (VH), fibrovascular proliferation (FVP) and traction retinal detachment (TRD). Based on the severity of PDR, patients with coexisting NV, VH, FVP and/or TRD were classified as TRD, patients with NV, VH and/or FVP were classified as FVP, and patients with NV and VH were classified as VH, respectively.

Patients were excluded from the study if they had received a vitreous injection of an anti-VEGF agent, or had been administered a sub-Tenon or intravitreal triamcinolone acetonide injection within the previous 6 months. In addition, patients diagnosed with neovascular glaucoma or who received PRP and underwent focal photocoagulation within 3 months were excluded. Furthermore, aphakic patients were also excluded.

Patients were divided into two groups. The first group, which contained subjects who underwent a vitrectomy for PDR and then progressed after the PRP for pre-PDR or non-severe PDR, was defined as the preoperative group. The preoperative PRP procedure was previously performed using a spot size of 400–500 μm on the retinal surface, 532 nm, 0.2 ms, 150–200 mW, and 1200–1500 spots divided over three to four times in the preoperative group.

The second group, which was defined as the intraoperative group, included a small number of patients who required a vitrectomy for a vitreous hemorrhage and proliferative membrane that developed following focal photocoagulation for a non-perfusion area. The preoperative focal photocoagulation procedure was previously performed according to the method reported for selective photocoagulation in order to reduce the developing ratio from the pre-PDR to PDR [19]. Coagulation spots were adjusted to 400–500 μm on the retinal surface, with the procedure performed on the non-perfusion area that was confirmed by the fluorescein angiography. The number of coagulation spots ranged from almost 100 to 300. Nearly all of the patients in the intraoperative group were not able to undergo PRP due to the presence of vitreous hemorrhage at the time of their initial visit.

### Surgical methods

Undiluted vitreous fluid was collected from PDR patients (130 eyes) at the time of the vitrectomy that was performed at Hirosaki University Hospital using a previously described method [8]. Briefly, vitreous fluid (approximately 0.3 ml) was obtained from each eye prior to staring the fluid irrigation during the initial stage of the vitrectomy procedure. Vitreous samples were immediately cooled on ice in a dark container for approximately 1 to 2 h and then frozen at − 80 °C until analysis.

Triamcinolone acetonide was applied during the vitrectomy order to visualize the vitreous. For patients without posterior vitreous detachment, we manually created a posterior vitreous detachment by suction with the vitreous cutter that was used to perform the vitrectomy procedure. We also tried to remove as much of the peripheral vitreous as possible. The membrane was completely removed in patients with fibrovascular membrane. While endophotocoagulation was performed as PRP in the intraoperative group (532 nm, 120 mW, 200 ms, 1800–2200 spots), endophotocoagulation was only performed in the peripheral retina in the preoperative group (532 nm, 120 mW, 200 ms, 100–500 spots). For this case series, we used a 3 port-vitrectomy system in conjunction with the Constellation Vision System that was manufactured by Alcon (Hünenberg, Switzerland).

For phakic eye patients, phacoemulsification and aspiration were performed after collecting the vitreous fluid sample, with an intraocular lens then fixed in the bag after completing the vitrectomy procedure. In pseudophakic eyes (7 eyes in the preoperative group and 1 eye in the intraoperative group), the intraocular lens was preserved. In cases where retinal tears developed during the surgery and were associated with the retinal detachment around it, the vitreous cavity was filled with 20% SF6 at the end of the surgery. Thereafter, patients remained in a face-down position for 1 week after the surgery.

Background clinical data for the preoperative conditions were obtained from each of the patient’s medical records.

### Quantitative analysis of cytokines

We measured the concentrations of inflammatory cytokines by the method previously described [8, 20–22]. Vitreous fluid was diluted fourfold with the dilution solution provided by a Bio-Plex^®^ beads array kit (Bio-Rad Laboratories, Hercules, CA, U.S.A.). For sample preparations, samples were centrifuged at 10,000*g* for 5 min after vortex agitation. A total of 50 µl of supernatant was used for the cytokine assay in accordance with the manufacturer’s instructions. For the Bio-Plex^®^ kit, beads separated by color for each target cytokine conjugated with primary antibodies against the target cytokine were obtained commercially. We measured the following 27 cytokines: IL-1β, IL-1 receptor antagonist (IL-1ra), IL-2, IL-4, IL-5, IL-6, IL-7, IL-8 [chemokine *C*-*X*-*C* motif ligand (*CXCL8*)], IL-9, IL-10, IL-12, IL-13, IL-15, IL-17, eotaxin [chemokine *C*–*C* motif ligand (*CCL11*)], basic fibroblast growth factor (bFGF), granulocyte-colony-stimulating factor (G-CSF), granulocyte/macrophage-colony-stimulating factor (GM-CSF), interferon (IFN)-α, IFN-γ, MCP-1 (*CCL2*), MIP-1α (*CCL3*), MIP-1β (*CCL4*), IP-10 (*CXCL10*), platelet-derived growth factor (PDGF), regulated upon activation, normal T cell expressed and secreted (RANTES, *CCL5*), and VEGF. The measurements of these cytokines were performed using 96-well assay plates and reagent kits according to the procedure provided by the manufacturer.

### Evaluation of postoperative inflammation

The severity of postoperative inflammation was analyzed retrospectively using medical records obtained from the consultation on the day after surgery. The presence of fibrin deposition in the anterior chamber or vitreous cavity was confirmed, and then compared between the preoperative and the intraoperative groups. For the examination, postoperative visual acuity, preoperative acuity as well as acuity at 6 months after surgery were compared between the groups. Complications that necessitated a reoperation within 1 year after surgery were considered to be postoperative and compared between the groups.

### Statistical analysis

The levels of various cytokines including VEGF are presented as means ± standard deviations. We performed Welch T test to compare cytokine expression between the preoperative group and the intraoperative group.

Chi-square test and Student T test were used to evaluate statistical differences in preoperative general conditions, preoperative ophthalmological conditions, rate of postoperative fibrin deposition, pre- and postoperative best-corrected visual acuities (logarithm of the minimum angle of resolution, logMAR), and postoperative complications between the groups. For this calculation, the logMAR for counting fingers was defined as 2.0, while hand motion and light perception were defined as 3.0.

## Results

Among the 130 eyes of the PDR patients, 67 eyes of 60 patients underwent PRP prior to surgery in the preoperative group, while 63 eyes of 46 patients underwent PRP during surgery in the intraoperative group. There were no significant differences regarding the preoperative age, sex, and diabetes control status between the groups. However, insulin treatment for diabetes was significantly more predominant in the preoperative as compared to the intraoperative group (Table 1).

**Table 1:** Preoperative general conditions of the preoperative and intraoperative groups

	Preoperative group 60 cases 67 eyes	Intraoperative group 46 cases 63 eyes
Age (years old)	56.6 ± 14.6	53.1 ± 13.3^1#^
Gender (male/female)	32 (53%)/28 (47%)	26 (57%)/20 (43%)^2#^
DM control level (HbA1c^a^ %)	7.8 ± 1.9	7.9 ± 2.2^3#^
DM therapy (diet/drug/insulin)	0 (0%)/21 (35%)/39 (65%)	6 (13%)/16 (35%)/24 (52%)^4#^

^1#^*p *= 0.16, unpaired *t*-test

^2#^*p *= 0.74, Chi-square test

^3#^*p *= 0.34, unpaired *t*-test

^4#^*p *= 0.015, Chi-square test

^a^HbA1c (NGSP)

PRP was completed before surgery in all 67 eyes in the preoperative group. In the intraoperative group, photocoagulation was partially performed in 8 (14.5%) and not performed in 55 (85.5%) eyes. The comparison of the the severity of the diabetic retinopathy that required vitrectomy showed that there were no significant differences for the ratios of retinal neovascularization, vitreous hemorrhage, fibrovascular proliferation, or the traction retinal detachment between the groups (Table 2).

**Table 2:** Preoperative ophthalmological conditions of the preoperative and intraoperative groups

	Preoperative group 60 cases 67 eyes	Intraoperative group 46 cases 63 eyes
Photocoagulation (PRP/focal PC/non PC)	67 (100%)/0 (0%)/0 (0%)	0 (0%)/8 (14.5%)/55 (85.5%)^1#^
Retinopathy condition (NV/VH/FVP/TRD)	5 (7%)/35 (52%)/23 (34%)/4 (6%)	4 (6%)/23 (37%)/21 (33%)/15 (24%)^2#^
Lens condition (phakia/pseudophakia)	60 (89.6%)/7 (10.4%)	63 (98.4%)/(1.6%)^3#^

*NV* retinal neovascularization, *VH* vitreal hemorrhage, *FVP* fibrovascular proliferation, *TRD* traction retinal detachment

^1#^p < 0.001, ^2#^p = 0.06, ^3#^p = 0.08, Chi-square test

Analysis of the intravitreal cytokines revealed there were no significant differences for the level of VEGF between the preoperative (4885.11 ± 6052.57 pg/ml) and intraoperative (5313.19 ± 6679.82 pg/ml) groups (p = 0.703). In contrast, there was a significantly higher IL-6 level in the intraoperative versus the preoperative group (2813.24 ± 6022.22 vs. 1248.55 ± 1442.20 pg/ml; Welch’s t-test, p = 0.048). Similarly, there was also a significantly higher IL-7 level in the intraoperative group (183.16 ± 216.86 vs. 140.23 ± 160.16; p = 0.024). There were no significant differences observed between the groups regarding the levels of the inflammatory cytokines (Table 3).

**Table 3:** Intravitreal cytokine levels in the preoperative and intraoperative groups

	Preoperative group	Intraoperative group	p value
IL-1β	33.36 ± 25.6	47.82 ± 58.68	0.077
IL-1Ra	52.18 ± 74.75	51.94 ± 57.84	0.984
IL-2	39.43 ± 28.49	49.49 ± 52.58	0.185
IL-4	46.66 ± 33.37	55.48 ± 61.06	0.316
IL-5	16.38 ± 15.17	36.88 ± 119.00	0.179
IL-6	1248.55 ± 1442.20	2813.24 ± 6022.22	0.048
IL-7	140.23 ± 160.16	183.16 ± 216.86	0.024
IL-8	2484.05 ± 3815.29	2931.02 ± 5054.09	0.572
IL-9	273.66 ± 253.88	291.79 ± 231.32	0.671
IL-10	691.81 ± 1124.22	669.53 ± 1378.81	0.920
IL-12	167.36 ± 287.64	191.15 ± 315.35	0.655
IL-13	263.98 ± 399.19	310.25 ± 547.07	0.649
IL-15	893.40 ± 417.00	995.13 ± 791.16	0.366
IL-17	269.37 ± 238.59	259.26 ± 240.32	0.810
IP-10	7242.70 ± 6765.15	8698.26 ± 8853.63	0.297
Eotaxin	58.30 ± 44.23	68.79 ± 71.69	0.323
FGF basic	369.40 ± 290.56	374.41 ± 303.43	0.924
G-CSF	62.43 ± 49.65	61.89 ± 49.48	0.074
GM-CSF	318.25 ± 360.94	364.32 ± 492.66	0.544
IFN-γ	31.98 ± 25.41	40.84 ± 47.92	0.195
MCP-1	12,935.94 ± 9333.96	13,931.40 ± 9392.86	0.549
MIP-1α	172.89 ± 189.55	177.05 ± 208.55	0.452
MIP-1β	1942.38 ± 2209.78	1842.21 ± 2224.01	0.797
PDGF bb	140.66 ± 129.86	153.77 ± 218.97	0.681
RANTES	163.42 ± 271.90	260.72 ± 796.88	0.360
TNF-α	215.16 ± 125.21	239.06 ± 173.96	0.373
VEGF	4885.11 ± 6052.57	5313.19 ± 6679.82	0.703

Welch t test

**Fig. 1:**
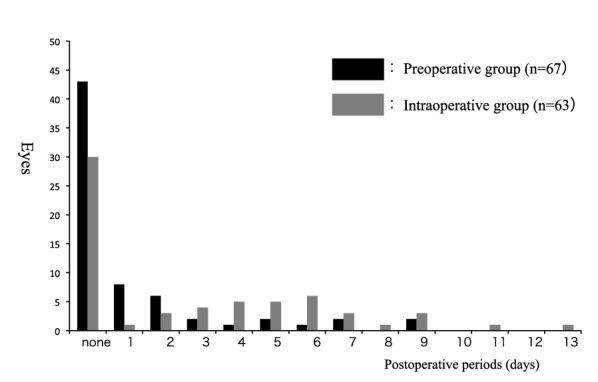
Eyes with fibrin deposition in the preoperative and intraoperative groups. Eyes with fibrin deposition in the preoperative group (black) and in the intraoperative group (gray) were observed in the postoperative periods

**Fig. 2:**
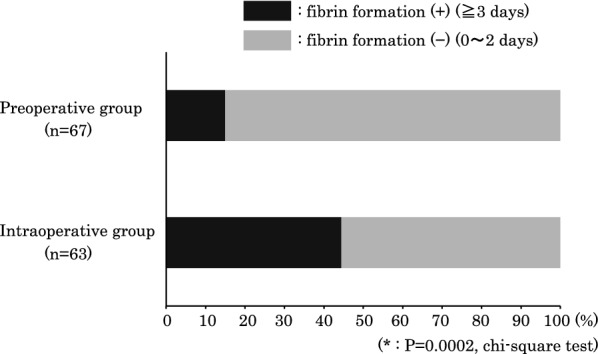
Rate of fibrin deposition in the preoperative and intraoperative groups. The rate of eyes with fibrin formation at and after 3 days following the surgery in the intraoperative group was higher than that observed in the preoperative group

Figures 1 and 2 show the results of examinations of the fibrin deposition in the anterior chamber and vitreous cavity, which were used for the evaluation of the postoperative inflammation. The number of eyes with and without fibrin deposition, and days until disappearance of deposition in each group are shown in Fig. 1. Although not common, eyes in which fibrin deposition remained for a long period were noted in the intraoperative group. As shown in Fig. 2, the number of eyes in which fibrin was observed at 3 days postoperatively was significantly smaller in the preoperative group (14.9%) as compared to the intraoperative group (44.4%) (p = 0.0002, Chi-square test).

Table 4 shows the changes in the visual acuity before and after surgery, as well as the postoperative complications in the two groups. In the preoperative group, pre- and postoperative logMAR acuity was 1.47 ± 0.16 and 0.69 ± 0.09, respectively, indicating there was significant improvement (p = 2.15 × 10^−5^, paired t-test). In contrast, there was no significant difference between before and after surgery for the logMAR acuity in the intraoperative group (0.19 ± 0.18 vs. 0.69 ± 0.14, p = 0.121, paired t-test).

**Table 4:** Postoperative visual acuities and postoperative complications in the preoperative and intraoperative groups

	Preoperative group (67 eyes)	Intraoperative group (63 eyes)
Visual acuity (logMAR)		
Preoperative VA	1.47 ± 0.16^1※^	0.91 ± 0.18^2※^
Postoperative VA (6 months)	0.69 ± 0.09^1※^	0.69 ± 0.14^2※^
Postoperative complications		
None	62 eyes (92.5%)	60 eyes (95.2%)
Vitreous hemorrhage	3 eyes (4.5%)	2 eyes (3.2%)^a^
Retinal detachment	2 eyes (3.0%)	1 eye (1.6%)
Neovascular glaucoma	0 eye (0.0%)	1 eye (1.6%)^a^

*VA* visual acuity

^1※^p = 2.15 × 10^−5^, ^2※^p = 0.121, paired-T test

^a^Both of vitreous hemorrhage and neovascular glaucoma occurred in one eye

Regarding postoperative complications, vitreous hemorrhage was observed in 3 (4.5%), retinal detachment in 2 (3.0%), and there were no complications in 62 (92.5%) eyes in the preoperative group. In the intraoperative group, vitreous hemorrhage was noted in 2 (3.2%), retinal detachment in 1 (1.6%), and neovascular glaucoma in 1 (1.6%), while there were no complications seen in 60 (95.2%) eyes. Among these complications in the intraoperative group, there was also 1 eye that developed vitreous hemorrhage and which was later complicated with neovascular glaucoma. There was no significant difference regarding the incidence of postoperative complications between the groups (Table 4).

## Discussion

Retinal photocoagulation techniques, especially PRP, are extensively used for patients with diabetic retinopathy in order to prevent progression and avoid resultant loss of vision [3, 4]. It has been recently reported that PRP using a conventional argon laser was more effective for PDR treatment as compared to that observed when using a pattern scan laser [23]. Moreover, there was a lower incidence of worsening vision reported for this method as compared to vitrectomy [24]. Thus, PRP is now considered to be an important treatment modality for PDR cases. Furthermore, other studies have found that PRP combined with a retinal injection of an anti-VEGF agent was more effective than the single application of PRP [5]. In addition, patients treated with photocoagulation combined with triamcinolone acetonide [6] have also been reported to have fewer incidences of macular edema as a postoperative complication. As these findings demonstrated the importance of photocoagulation, as well as its use as a treatment, recent investigations have begun to examine intraocular VEGF and inflammatory cytokines.

In the present study, our results showed that there was no significant difference in intravitreal VEGF between the intraoperative and the preoperative groups. Generally, since PRP has been shown to reduce the ischemic areas in the retina, it is thought that intravitreal VEGF decreases after PRP. Among the patients that have undergone PRP, some of these subjects developed proliferative changes in the retina and thus, needed to undergo vitrectomy. Furthermore, there was a gradual increase in the intravitreal VEGF concentration that also observed after the PRP. Therefore, we speculated that the intravitreal VEGF levels in the preoperative group cases might also be as high as those seen in the patients without preoperative PRP in the intraoperative group.

In the present study, we retrospectively compared the results of PDR patients who underwent a vitrectomy as the PDR progressed (even after undergoing PRP (preoperative group)), with the results for patients who underwent a surgical procedure without prior PRP (intraoperative group). Our findings revealed that among the examined intravitreal cytokines, the levels of IL-6 and IL-7 were significantly higher in the intraoperative versus the preoperative group. In contrast, we found no significant differences for the levels of the other cytokines, including VEGF, which is thought to be associated with PDR severity or the state of retinal ischemia. We also found no differences for IL-8, an inflammatory cytokine or MCP-1, which has been reported to be elevated in PDR patients. Although it has also been reported that a high level of IL-6 is immediately seen after PRP [25], this was not observed in our present cases. This was likely due to the fact that PRP was performed at a relatively long period prior to the surgery in the preoperative group.

The remarkable fibrin formations and the minimal fibrin formations were counted in the present study in order to determine the characteristics of the diabetic retinopathy inflammation. Therefore, the ratio of the postoperative fibrin formation, which was 44.4% in the intraoperative group, was relatively higher than that reported in other previous studies, for example, the 22% reported in study by Diolaiuti et al. [26].

The present study was not randomized but retrospective, thus one could agree that differences in the activity of retinopathy at the time of the vitrectomy are possible. The evaluation of the postoperative inflammation severity demonstrated that the intraoperative group exhibited a tendency for more prolonged inflammation as compared to that observed for the preoperative group. These results are in agreement with the results that showed that the intravitreal level of IL-6, which is an inflammatory cytokine, was higher in the intraoperative group. Furthermore, it is likely that the postoperative inflammation was severe in these patients, as the PRP was performed during the vitreous surgical procedure. Thus, it is reasonable to consider that postoperative inflammation could be increased due to the elevated IL-6 level as well as due to the aggressiveness of the surgical invasion associated with the intraoperative PRP.

The inflammatory cytokine, IL-6, is known to be present in significantly higher concentrations in the vitreous fluid and in the aqueous humor of the anterior chamber in DR patients as compared to that observed in healthy individuals. However, the association of IL-7 with clinical conditions related to DR has yet to be definitively investigated and thus, the potential association currently remains unclear. Even so, a previous report did note that the levels of not only VEGF, but also IL-6, IL-7, MCP-1, and TNF-α in the aqueous humor of the anterior chamber showed a greater decrease after an anti-VEGF intravitreal injection as compared to that observed before the injection, and which was shown to be associated with DR [27]. There were no significant differences in postoperative complications between the preoperative and intraoperative groups. Although the severity of the postoperative inflammation varied, it was not great enough to result in differences in the frequency of complications that would thereby require a reoperation. As for postoperative visual acuity, there were no significant differences observed for preoperative acuity in the intraoperative group, whereas that was a significant improvement in the acuity after the surgery in the preoperative group. In the patients in the preoperative group that had previously undergone PRP, their vision remained relatively stable and was maintained even with the development of retinal neovascularization. However, some of the preoperative group cases might subsequently have needed to undergo a vitrectomy, as vitreous hemorrhage was induced and the proliferative membrane emerged as a result of posterior vitreous detachment. Nevertheless, even in these types of cases, we speculated that their postoperative vision could have been easily restored, as these types of patients are known to have a lower amount of irreversible macular change.

While we cannot conclude that difficulties in achieving a better postoperative vision in patients without PRP prior to surgery was due to severe postoperative inflammation, it is likely that severe inflammation does occur postoperatively in cases without preoperative PRP. Thus, one way to potentially prevent this from occurring is to administer a triamcinolone injection into the vitreous cavity or sub-Tenon at the completion of surgery, along with the concomitant postoperative intensified use of steroid eye drops, which are considered to be effective in these cases.

## Data Availability

Not applicable.
